# Lifetime Impact of Cow’s Milk on Overactivation of mTORC1: From Fetal to Childhood Overgrowth, Acne, Diabetes, Cancers, and Neurodegeneration

**DOI:** 10.3390/biom11030404

**Published:** 2021-03-09

**Authors:** Bodo C. Melnik

**Affiliations:** Department of Dermatology, Environmental Medicine and Health Theory, University of Osnabrück, Am Finkenhügel 7a, D-49076 Osnabrück, Germany; melnik@t-online.de; Tel.: +49-5241-988-060

**Keywords:** acne vulgaris, amino acids, cancer, diabetes mellitus, growth, milk, milk exosomal microRNAs, mortality, mTORC1, neurodegeneration

## Abstract

The consumption of cow’s milk is a part of the basic nutritional habits of Western industrialized countries. Recent epidemiological studies associate the intake of cow’s milk with an increased risk of diseases, which are associated with overactivated mechanistic target of rapamycin complex 1 (mTORC1) signaling. This review presents current epidemiological and translational evidence linking milk consumption to the regulation of mTORC1, the master-switch for eukaryotic cell growth. Epidemiological studies confirm a correlation between cow’s milk consumption and birthweight, body mass index, onset of menarche, linear growth during childhood, acne vulgaris, type 2 diabetes mellitus, prostate cancer, breast cancer, hepatocellular carcinoma, diffuse large B-cell lymphoma, neurodegenerative diseases, and all-cause mortality. Thus, long-term persistent consumption of cow’s milk increases the risk of mTORC1-driven diseases of civilization. Milk is a highly conserved, lactation genome-controlled signaling system that functions as a maternal-neonatal relay for optimized species-specific activation of mTORC1, the nexus for regulation of eukaryotic cell growth, and control of autophagy. A deeper understanding of milk´s impact on mTORC1 signaling is of critical importance for the prevention of common diseases of civilization.

## 1. Introduction

The health-related effects of cow milk consumption by humans has been the focus of recent epidemiological research [1,2]. Milk is a substantial component of nutrition in Western industrialized countries. For instance, the annual per capita milk consumption in Germany was 49.5 L in 2019 [3]. Milk consumption is even higher in Scandinavian countries. The annual per capita milk consumption in Sweden declined from 2007 to 2018, from 130.5 L to 98.2 L [4]. In contrast, milk consumption in Asian countries is much lower. However, China’s per capita milk consumption increased in recent years. In 2019, Chinese consumed on average 12.5 kg of milk and dairy products per person [5].

There is accumulating evidence that milk, the secretory product of mammary glands promoting growth and anabolism of newborn mammals, is not a simple food, but a signaling system activating the nutrient- and growth factor-sensitive kinase mechanistic target of rapamycin complex 1 (mTORC1) [6,7].

mTORC1 is an evolutionary conserved Ser/Thr protein kinase that senses multiple upstream stimuli to control cell growth, metabolism, and autophagy. mTOR is the catalytic subunit of mTOR complex 1 (mTORC1). A significant amount of research has uncovered the signaling pathways regulated by mTORC1, and the involvement of these signaling cascades in human diseases, such as cancer, diabetes, and aging [8].

It is important to realize that large-scale consumption of fresh cow’s milk is a novel human behavior introduced by the availability of pasteurization and refrigeration technology [9]. Since the Neolithic revolution, over 10,000 years of preferentially fermented milk (yogurt, cheese) were the predominant milk products consumed by humans. Recent evidence has suggested that microbial fermentation of milk attenuates milk-mediated mTORC1 signaling, extensively reviewed elsewhere [9].

It is the intention of this review to present epidemiological and translational evidence that links milk consumption to mTORC1-driven pathologies and diseases of civilization. To understand milk´s impact on mTORC1 activation, a brief introduction of milk-derived signals promoting mTORC1 signaling will be provided first. Then, milk´s effects on mTORC1 signaling beginning from fetal growth, childhood, puberty, adolescence, and senescence will be presented.

## 2. Milk: A Relay for mTORC1-Activation of the Milk Recipient

Human breastmilk is the physiological and exclusive secretory product of the human mammary gland, supporting postnatal growth and appropriate metabolic programming of the newborn infant. Human breastmilk is evolutionarily adapted to meet the optimal species-specific growth requirements of the infant, resulting in the World Health Organization (WHO) recommendation of exclusive breastfeeding for six months [10]. Whereas formula feeding is an artificial attempt to imitate human breastmilk, recent pediatric research acknowledges the advantages of breastfeeding for approaching desirable growth trajectories and favorable metabolic long-term outcomes [11,12]. Surprisingly, when human milk composition is discussed in relation to infant growth, milk macronutrients, hormones, milk oligosaccharides, micronutrients, microbiota, and other bioactive components [13], these compounds have not been related to the cell´s central activator of growth and anabolism, the kinase mTORC1 [14,15,16,17,18,19]. However, to understand milk-mediated growth and anabolism, milk-signaling interaction with mTORC1 of the milk recipient has to be appreciated. Milk consumption activates five major pathways stimulating mTORC1 via (1) growth factors, including growth hormone (GH), insulin, and insulin-like growth factor 1 (IGF-1), (2) amino acids, especially branched-chain amino acids (BCAAs), (3) milk fat-derived palmitic acid, (4) the milk sugar lactose (β-D-galactopyranosyl-(1→4)-D-glucose, and (5) epigenetic modifiers, especially milk exosome (MEX)-derived micro-ribonucleic acids (miRs).

### 2.1. Milk-Induced Growth Factor Signaling

#### 2.1.1. Growth Hormone and Insulin-Like Growth Factor-1

**Figure 1:**
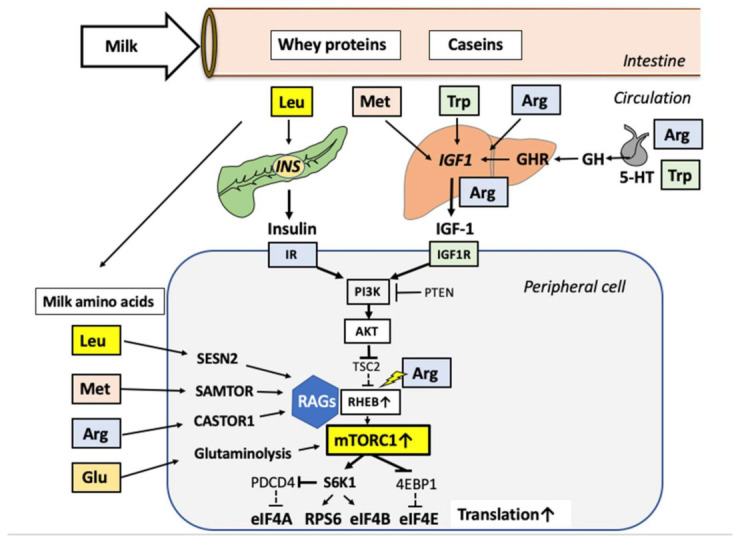
Model of milk amino acid signaling activating mTORC1 directly via amino acid-RAG interaction and insulin/IGF-1/PI3K/AKT signaling activating RHEB at the lysosomal membrane. Abbreviations: GH: growth hormone; GHR: growth hormone receptor; 5-HT: 5-hydroxy-tryptamine; *INS*: insulin gene; IR: insulin receptor; *IGF1*: IGF-1 gene; IGF-1: insulin-like growth factor 1; IGF1R: IGF-1 receptor; PI3K: phosphoinositide-3-kinase; PTEN: phosphatase and tensin homolog; AKT: Akt kinase (protein kinase B); TSC2: tuberin; SESN2: sestrin 2; SAMTOR: S-adenosylmethionine sensor upstream of mTOR; CASTOR1: cellular arginine sensor for mTORC1; RHEB: ras homolog enriched in brain. RAG: ras-related GTP binding protein; mTORC1: mechanistic target of rapapmycin complex 1; PDCD4: programmed cell death 4, S6K1: ribosomal protein S6 kinase 1; 4EBP1: eukaryotic translation initiationfactor 4E-binding protein 1; eIF4A: eukaryotic translation initiation factor 4A; RPS6: ribosomal protein S6; eIF4B: eukaryotic translation initiation factor 4A; eIF4E: eukaryotic translation initiation factor 4A; Leu: leucine; Met: methionine; Arg: arginine; Glu: glutamine; Trp: tryptophan.

Milk consumption enhances growth hormone (GH) levels in children and peak GH levels in adults [20,21], as well as circulating IGF-1 levels in children and adults [20,21,22,23,24,25,26]. IGF-1 is a component of human and bovine milk [27,28,29]. Notably, the amino acid sequence of human and bovine IGF-1 are identical [30]. The GH–IGF-1 axis not only plays a key role for the physiological growth during childhood [30,31,32], but is also involved in milk production of dairy cows [33]. Administration of bovine GH (banned in the European Union) to dairy cows results in increased IGF-1 milk levels [34]. It is noteworthy to mention that it is not the oral uptake of bovine GH and bovine IGF-1 in milk that increases serum IGF-1 levels of milk consumers, but predominantly milk´s inductive effect on hepatic IGF-1 synthesis [20,29]. Milk-induced increases in GH signaling via the GH receptor (GHR) and milk protein-derived amino acids, especially tryptophan, methionine, and arginine, enhances hepatic IGF-1 synthesis and secretion [20,35,36,37,38,39,40,41], resulting in enhanced IGF-1 mediated linear growth [42,43,44]. Tryptophan, a major component of milk proteins, is the precursor of serotonin (5-hydroxytryptamine, 5-HT), which, via 5-HT2 receptors, stimulates hypothalamic growth hormone releasing hormone (GHRH) release and pituitary GH secretion increasing serum GH levels [45]. GH binding to hepatic GH receptor (GHR) is the major mechanism increasing circulatory levels of IGF-1 [46,47]. IGF-1, after binding to IGF-1 receptor (IGF1R), activates the phosphoinositide-3 kinase (PI3K)-AKT pathway, which phosphorylates tuberin (TSC2) resulting in dissociation of TSC2 from the lysosomal membrane. This results in the activation of RAS homolog enriched in the brain (RHEB), which finally activates mTORC1 [16,19,48,49,50,51,52] (Figure 1).

#### 2.1.2. Insulin

Insulinotropic BCAAs of milk are released by milk protein hydrolysis in the intestine and induce postprandial hyperinsulinemia. That is why the insulinemic index of milk is three times higher than milk’s glycemic index [53,54]. Predominantly, whey protein-derived amino acids released after fast intestinal hydrolysis are responsible for the insulinemic effect of milk [55,56,57,58,59]. Insulin and IGF-1 synergistically activate PI3K-AKT-mTORC1 signaling, growth, and anabolism [49,51,60,61,62,63,64,65].

### 2.2. Milk-Derived Amino Acids

Among other animal or plant proteins, milk protein represents the richest source of BCAAs (Table 1). Milk and casein contain high amounts of methionine. In comparison to meat, whey proteins contain highest amounts of leucine [66,67]. In comparison to beef (glutamine 4.75 g/100 g protein), milk protein has a high glutamine content (8.1 g/100 g protein) [68]. Glutamine via the glutaminolysis pathway also activates mTORC1 [69,70]. In comparison to plant and meat proteins, milk proteins (and especially whey proteins) exhibit an accelerated intestinal hydrolysis with increased postprandial concentrations of milk protein-derived amino acids that activate mTORC1 [59,71,72].

Major amino acids of milk proteins, such as leucine, arginine, and methionine are sensed via sestrin 2 (SESN2), cellular arginine sensor for mTORC1 (CASTOR1), and S-adenosylmethionine sensor upstream of mTOR (SAMTOR), respectively. They orchestrate mTORC1 activation through the well-characterized RAG GTPase signaling pathways [73,74,75,76,77,78,79,80,81,82,83,84,85,86,87,88]. Binding between active RAG GTPase complexes and Raptor recruit the complex to the lysosomal membrane [73,74,75,76,77,78,79,80,81,82,83,84,85,86,87,88]. Glutamine activates mTORC1 through a RAG GTPase-independent mechanism that requires ADP-ribosylation factor 1 (ARF1) [89]. Leucyl-tRNA synthetase (LRS) is another amino acid-dependent regulator of TORC1 [90,91,92]. LRS plays a critical role in amino acid-induced mTORC1 activation by sensing intracellular leucine concentration and initiating molecular events leading to mTORC1 activation. LRS directly binds to RAG GTPase, the mediator of amino acid signaling to mTORC1, in an amino acid-dependent manner and functions as a GTPase-activating protein (GAP) for RAG GTPase to activate mTORC1 [92]. In addition, LRS functions as a leucine sensor for the activation of the class III PI3K Vps34 that mediates amino acid signaling to mTORC1 by regulating lysosomal translocation and activation of the phospholipase PLD1 [93]. Direct visualization of leucine sensing and LRS translocation to the lysosome was related to a crosstalk between leucine sensing, LRS translocation, RAGD interaction, and mTORC1 activation [94]. Recent evidence indicates a role of LRS1 in glucose-dependent control of leucine usage. Upon glucose starvation, LRS1 was phosphorylated by unc-51 like autophagy activating kinase 1 (ULK1) at the residues crucial for leucine binding. The phosphorylated LRS1 exhibits decreased leucine binding, which may inhibit protein synthesis and help save energy [95].

Furthermore, arginine interferes with the TSC–RHEB complex relieving allosteric inhibition of RHEB by TSC [96]. Thus, arginine cooperates with growth factor signaling, which further promotes dissociation of TSC2 from lysosomes and activation of mTORC1 [96].

According to current consensus, mTORC1 is only activated when both RAG and RHEB GTPase activation pathways are fully activated, neither being sufficient in isolation [87]. RHEB and RAGs, the final activators of growth factor and amino acids signaling pathways, come together at the lysosome to activate mTORC1 [71,72,73,74,75,76,77,78,79,80,81,82,83,84,85,86,87,88,97].

### 2.3. Milk Lipids

The predominant fatty acid of milk triacylglycerols (TAGs), transported via milk fat globules (MFGs), is the saturated fatty acid palmitic acid (C16:0) [98,99,100]. MFG is a rapid conveyor of energy through its TAG core [101]. Palmitic acid, which after intestinal TAG hydrolysis and re-esterification into chylomicrons serves as an energy source, when catabolized by mitochondrial β-oxidation generates ATP [102,103]. ATP via inhibition of AMP-activated protein kinase (AMPK) activates mTORC1 at the lysosome [104,105,106]. Findings in skeletal muscle cells indicate that palmitate activates mTORC1/p70S6K signaling by AMPK inhibition and phosphorylation of Raptor [107]. Recent evidence supports the involvement of palmitic acid in mTORC1 activation at the lysosome [108,109].

Palmitate activates mTORC1 by enhancing the recruitment of mTOR onto lysosomal membranes, which is inhibited by co-incubation with oleate or eicosapentaenoic acid [108]. Recent evidence indicates that protein palmitoylation is potentially involved in palmitate-induced mTORC1 activation, whereas 2-bromopalmitate, a protein palmitoylation inhibitor, ameliorated palmitate-triggered mTORC1 activation [110]. Furthermore, MFG membrane proteins, predominantly MFG-E8, promote cell proliferation through the PI3K/AKT/mTORC1 signaling pathway [111,112].

### 2.4. Lactose

After the breastfeeding period, the mucosal expression of lactase, the intestinal enzyme hydrolyzing lactose into glucose and galactose is downregulated in all mammals with the exception of Neolithic humans, who developed *LCT* mutations allowing persistent lactase expression [113]. The lactose content of milk makes up around 2–8% by weight. Lactose hydrolysis provides glucose and galactose, which both activate mTORC1.

During glucose abundance and glycolysis, sufficient cellular energy is produced in the form of ATP, which suppresses AMPK activity. Under conditions of low energy, AMPK phosphorylation of TSC2 and Raptor attenuates mTORC1 activity [114,115,116,117,118]. Via an AMP/ADP-independent mechanism the glycolytic intermediate fructose-1,6-bisphosphate (FBP) is sensed by aldolase, which binds to the v-ATPase on the lysosomal surface. In the absence of FBP, interactions between aldolase and the v-ATPase are altered, allowing formation of an AXIN-based AMPK-activation complex containing the v-ATPase, Ragulator, AXIN, LKB1, and AMPK, causing increased Thr172 phosphorylation and AMPK activation [119,120]. Thus, aldolase operates as a sensor for glucose availability that directly links glucose shortage to activation of AMPK [119].

Accumulating evidence from other experimental models supports the view that galactose via induction of oxidative stress activates mTORC1 [121,122,123]. Notably, galactose-induced overactivation of mTORC1 promotes senescence of neural stem cells and aging of mesenchymal stem cells [122,124,125]. The role of oxidative stress on mTORC1 is still a highly debatable subject. Of interest, various lactobacilli used in food and dairy fermentation increase NRF2 activation resulting in NRF2-induced sestrin expression, which attenuates mTORC1 activation [126,127].

Taken together, milk provides a variety of macronutrients that relay the appropriate, species-specific activation of mTORC1 [7]: (1) amino acids that induce growth factor signals (GH; insulin, IGF-1); (2) a well-balanced array of amino acids that communicate with amino acids sensors that activate mTORC1; (3) milk lipids, especially palmitic acid, which activates mTORC1; and (4) lactose and its hydrolysis products glucose and galactose that provide cellular energy and promote mTORC1 activation. This complex endocrine system has been shaped to perfection over millions of years of mammalian evolution [128,129,130]. As mammals rely on milk for the promotion of postnatal growth, the effectors provided by the lactation genome on the donor site and the milk sensors of the milk recipient have to interact in a synergistic fashion to fulfill milk´s biological function: the activation of mTORC1, the primary cell-autonomous nutrient sensor for growth and maturation in mammals [131].

### 2.5. Milk Exosomal MicroRNAs

**Figure 2:**
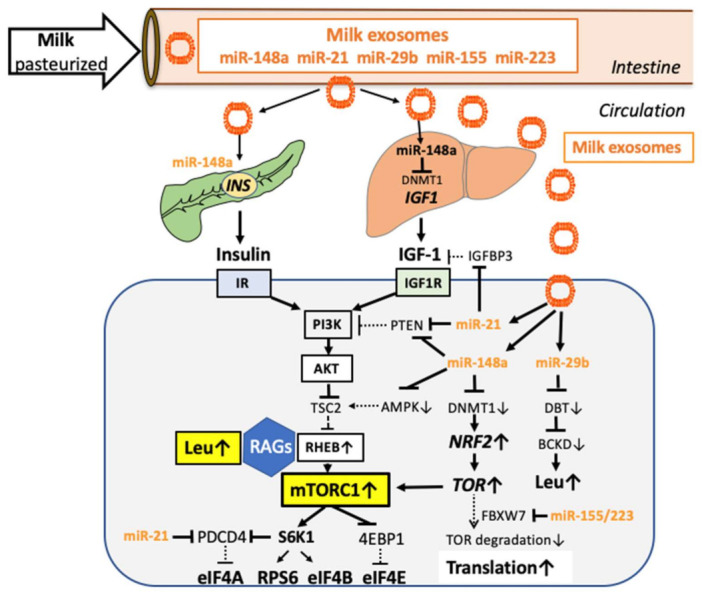
Model of milk miR-mediated epigenetic regulation increasing mTORC1 signaling. Milk-derived exosomal miRs enhance insulin/IGF-1/PI3K/AKT signaling, enhance intracellular levels of BCAAs, and promote mTOR expression. Abbreviations: miR: micro-ribonucleic acid; DNMT1: DNA methyltransferase 1; *INS*: insulin gene; IR: insulin receptor; *IGF1*: IGF-1 gene; IGF-1: insulin-like growth factor 1; IGFBP3: IGF binding protein 3; IGF1R: IGF-1 receptor; PI3K: phosphoinositide-3-kinase; PTEN: phosphatase and tensin homolog; AKT: Akt kinase (protein kinase B); AMPK: AMP-activated protein kinase; TSC2: tuberin; RHEB: ras homolog enriched in brain; Leu: leucine; RAG: ras-related GTP binding protein; mTORC1: mechanistic target of rapapmycin complex 1; PDCD4: programmed cell death 4, S6K1: ribosomal protein S6 kinase 1; 4EBP1: eukaryotic translation initiation factor 4E-binding protein 1; eIF4A: eukaryotic translation initiation factor 4A; RPS6: ribosomal protein S6; eIF4B: eukaryotic translation initiation factor 4B; eIF4E: eukaryotic translation initiation factor 4E; NRF2: nuclear factor erythroid 2-related factor 2; TOR: target of rapamycin; FBXW7: F-box and WD40 domain protein 7; DBT: dihydrolipoamide branched-chain transacylase; BCKD: branched-chain alpha-ketoacid dehydrogenase.

Pasteurized milk transfers bioavailable milk-derived exosomes (MEX) and their gene-regulatory microRNAs (miRs) [132,133,134,135,136,137]. Bovine and human MEX and their miRs resist degradative conditions in the gastrointestinal tract, reach the systemic circulation, and distribute in various tissues [134,138,139,140,141,142,143,144]. In fact, increasing evidence presented by studies in humans and animal models supports the view that MEX and their miRs are bioavailable and reach the systemic circulation [134,136,145,146,147], and modify gene expression of the milk recipient [132,148,149,150]. MEX miR-mediated changes of epigenetic regulation appear to be beneficial for growth and maturation of the infant [143,151,152,153,154,155,156,157,158], but may exert adverse health effects during long-term exposure associated with persistent overactivation of mTORC1 (Figure 2) [159].

#### 2.5.1. MiR-148a

MiR-148a is the most abundant miR in cow milk and MEX [132,160,161,162,163,164] and is highly conserved between mammals [165]. Notably, *MIR148A* is a domestication gene of dairy cattle increasing milk yield [166,167]. Milk miR-148a nucleotide sequences of humans and dairy cows are identical [132] (mirbase.org, accessed 16 February 2021), allowing miR-based cross-species communication between cattle and human milk consumers [168]. A major target of miR-148a is DNA methyltransferase 1 (DNMT1) [169] resulting in MEX-mediated suppression of DNMT1 expression [132,149], a key mechanism modifying postnatal epigenetic regulation activating mTORC1 signaling [150,153,170,171]. Impaired DNMT1-dependend promoter methylation increases the expression of various developmental genes including insulin (*INS*) [172], IGF-1 (*IGF1*) [173] and fat mass- and obesity-associated gene (*FTO*) [174,175,176,177], which all promote insulin/IGF-1-PI3K-AKT- and FTO/amino acid-mediated activation of mTORC1 [178,179].

FTO is a N6-methyladenosine (m6A) demethylase, which controls the expression of several components of the mTORC1 pathway [180,181,182,183]. Milk via miR-148a-, miR-21- and miR-29b-mediated suppression of DNMTs may promote CpG demethylation at intron 1 of FTO increasing FTO expression amplifying the m6A-regulated transcriptional machinery for postnatal growth [184]. DNMT1 inhibition upregulates the expression of nuclear factor erythroid 2-related factor 2 (NRF2) [185], a key transcription factor promoting the expression of mTOR (*MTOR*) [186]. MiR-148a also attenuates the expression AMP-activated protein kinase (AMPK) via targeting the catalytic subunit α1 of AMPK (*PRKAA1*) as well as the AMPK regulatory subunit γ2 (*PRKAG2*) [187] (targetscan.org, accessed 16 February 2021). AMPK directly phosphorylates at least two proteins to induce rapid suppression of mTORC1 activity, the TSC2 tumor suppressor, and the critical mTORC1 binding subunit Raptor [104,116]. In addition, miR-148a targets phosphatase and tensin homolog (PTEN) the upstream negative regulator of PI3K [149]. Thus, miR-148a, the most abundant miR of cow milk, epigenetically augments several checkpoints of growth factor- and amino acid signaling pathways that activate mTORC1.

#### 2.5.2. MiR-21

Bovine miR-21 is another abundant signature miR of cow milk [160] with nucleotide sequence homology to human miR-21 [188] (mirbase.org, accessed 16 February 2021). By use of RNase H2-dependent PCR, which distinguishes between bovine and human miRs with small variations in the nucleotide sequence, plasma concentrations of *Bos taurus* (bta)-miR-21-5p was >100% higher 6 h after commercial cow milk consumption of healthy human volunteers than before milk consumption strengthening the bioavailability of milk-derived miRs in human milk consumers [136]. In analogy to miR-148a, miR-21 attenuates the expression of DNMT1 [169], thus modifies epigenetic regulation. Importantly, miR-21 activates mTORC1, promotes growth and anabolism [6], and is regarded as an oncomir promoting sustained cell proliferation and cancer growth [189,190,191,192,193,194,195,196,197]. In particular, miR-21 inhibits key suppressors of the mTORC1 pathway such as IGF binding protein 3 (IGFBP3) [194], PTEN [189,190,191], and the inhibitor of translation initiation programmed cell death 4 (PDCD4) [190,192,193].

#### 2.5.3. MiR-155 and MiR-223

Further dominant immune-regulatory miRs of bovine milk are miR-155 and miR-223 [138,139,163,198,199]. MiR-155 also targets IGFBP3 [200] and PTEN [201]. MiR-155 and miR-223 suppresses mTOR degradation via targeting the expression of F-box and WD40 domain protein 7 (FBXW7) [202] (targetscan.org, accessed 16 February 2021), a key regulatory checkpoint that mediates ubiquitination-dependent degradation of mTOR [203].

#### 2.5.4. MiR-125b and MiR-30d

MiR-125b is another important bovine miR in milk, which withstands digestion under simulated gastrointestinal tract conditions [139,162,199]. MiR-30d belongs to the top 10 expressed miRs when parsing the sequence data, based on different species (buffalo, cow, pig, human, and panda milks) [132,147,204,205]. Notably, both miR-125b and miR-30d inhibit the expression of TP53, the guardian of the genome [206,207,208]. Recent evidence indicates that bovine MEX transfected with fluorophore (IRDye)-labeled miR-30d and miR-21 accumulated in murine placenta and embryos of C57BL/6 mice after oral gavage [209]. In accordance, MEX-associated and free human miR-30d was internalized by mouse embryos via the trophectoderm, resulting in an indirect overexpression of genes encoding for certain molecules involved in murine embryonic adhesion [210]. MEX miR-125b and miR-30d via targeting TP53 may represent another key mechanism of milk modifying mTORC1 signaling [211].

In particular, p53 induces the expression of a group of p53 target genes in the IGF-1/AKT and mTORC1 pathways, and all of these gene products negatively regulate the IGF-1/AKT and mTORC1 pathways in response to stress signals. They are IGFBP3 [212], PTEN [213,214,215,216], TSC2 [213], AMPK β1 [213], Sestrin1, and Sestrin2 [217]. With the exception of Sestrin2, which via leucine sensing also activates mTORC1 [218] and via AMPK activation that inhibits mTORC1 [217,219], all other p53 targets increase mTORC1 signaling [211].

#### 2.5.5. MiR-29b

MiR-29b is another important miR of commercial cow milk, which survives pasteurization and storage [133]. Bovine MEX miR-29b is taken up by intestinal epithelial cells via endocytosis [220]. After consumption of 0.25, 0.5, and 1.0 L of commercial milk, respectively, plasma levels of miR-29b increased after 6 h in a dose-dependent manner and modified blood monocyte gene expression [148]. In synergy with the DNA methylation-suppressing effects of miR-148a and miR-21, miR-29b also attenuates the expression of DNMT3A/B [221,222,223,224]. Thus, signature miRs of milk shape the epigenome and enhance the expression of developmental genes that increase mTORC1 signaling [153,170,171,184].

MiR-29b attenuates BCAA catabolism via targeting the mRNA for the dihydrolipoamide branched-chain transacylase (DBT), the E2-core subunit of branched-chain α-ketoacid dehydrogenase (BCKD) increasing cellular BCAA levels [225]. BCKD activity is regulated through the action of the complex-specific BCKD kinase that phosphorylates two serine residues in the E1α subunit and thereby inhibits BCKD. Notably, insulin stimulates BCKD kinase expression inhibiting BCKD increasing cellular BCAA levels [226,227,228,229,230,231]. Mechanistically, MEX miR-29b functions as an enhancer of insulin-mediated suppression of BCAA catabolism promoting mTORC1 activation at both the PI3K/AKT/TSC2/RHEB and the BCAA/RAG-Ragulator/RHEB pathway.

## 3. Milk-Induced Overactivation of mTORC1 and Diseases of Civilization

The impact of cow’s milk consumption in Western countries already begins during pregnancy, affecting the fetal growth period, accompanying the infant and childhood growth period, puberty, adulthood, and higher ages. Epidemiological and translational evidence will be presented that milk-induced overactivation of insulin/IGF-1 signaling combined with extensive supply of dairy-derived essential amino acids and milk-derived miRs overstimulates mTORC,1 promoting Western diseases of civilization [232,233].

### 3.1. Fetal Growth and Birthweight

The Danish National Birth Cohort shows an association between maternal milk consumption and birthweight [234], subsequently confirmed by further systematic reviews [235,236,237,238]. Increased trophoblast mTORC1 activity determines placental–fetal transfer of amino acids and glucose and thus fetal growth and birthweight [239,240,241,242,243,244]. Recent evidence underlines that mTORC1 signaling regulates the expression of trophoblast genes involved in ribosome and protein synthesis, mitochondrial function, lipid metabolism, nutrient transport, and angiogenesis, representing novel links between mTOR signaling and multiple placental functions critical for fetal growth and development [245]. Not only milk-derived BCAAs, but also bovine MEX and their miR cargo, delivered by oral gavage, reach the murine placenta [209]. Of note, fluorophore-labeled MEX, miR-21-5p, and miR-30d accumulate in murine placenta and embryos following oral MEX administration [209]. Remarkably, the size of litters born to dams fed a MEX- and RNA-depleted diet was 25–50% smaller than those born to MEX- and RNA-sufficient controls [209], pointing to a significant gene-regulatory contribution of MEX miRs for fetal growth. It is assumed that 60% of genes and their expression is regulated by miRs. In fact, increased human placental miR-21 levels correlate with the risk of fetal macrosomia [246,247]. Remarkably, cow’s milk consumption by humans during pregnancy, but not the intake of fermented milk products, increase birthweight [234,235], which underlines the mTORC1-activating and growth promoting effects of MEX. Thus, high milk consumption during pregnancy promotes mTORC1-driven fetal overgrowth [248]. In accordance, cafeteria and high-fat diets in nursing rats and mice modifies specific miR levels in milk [249,250]. Cow’s milk consumption during the lactation period may as well change the composition of milk miRs promoting postnatal growth.

### 3.2. Menarche, Height, Body Mass Index

The National Health and Nutrition Examination Survey (NHANES) [251] and the Tehran Lipid and Glucose Study [252,253] report an association between cow’s milk consumption and early menarche, a risk factor of breast cancer (BC) [254], which correlates to breast density [255]. NHANES also demonstrates an association between cow’s milk consumption and linear growth [42,256], well explainable by the increased somatotropic axis (GH/IGF-1) due to milk consumption [20,43,257]. NHANES also reports a milk-dependent increase of body mass index (BMI), predominantly in infants 2–4 years of age [258]. The increase of growth parameters and BMI by milk consumption points to an overactivation of mTORC1 by milk consumption, which is not observed with the consumption of fermented milk products [252,256].

### 3.3. Acne Vulgaris

Increased height and BMI during puberty correlates with a higher incidence of acne vulgaris [259,260,261,262], the most common inflammatory skin disease in industrialized countries, pointing to common accelerated growth trajectories in acne pathogenesis. The relationship between cow milk consumption and acne has been confirmed by recent meta-analyses [263,264,265]. In contrast, lactose-intolerant individuals, who generally avoid milk, exhibit a 50% lower frequency of acne compared to lactose-tolerant people [266]. Of concern, more severe acne during adolescence correlates with a higher risk of prostate cancer (PCa) and BC [267,268,269,270].

Acne is an IGF-1- and androgen-dependent disease of human sebaceous glands associated with sebaceous gland hyperplasia, increased and disturbed sebaceous lipogenesis, and enhanced proliferation of acro-infundibular keratinocytes (comedogenesis) [271,272]. Acne represents the prototype of an mTORC1-driven skin disease [273,274,275,276]. In fact, pathologically increased mTORC1 activity has been measured in epidermis and sebaceous glands of acne patients [277,278,279,280]. Thus, acne is regarded as the mTORC1-driven metabolic syndrome of the pilosebaceous follicle [281]. In accordance with states of overactivated mTORC1/S6K1 signaling, acne is often associated with insulin resistance [282,283,284,285]. A potential explanation is mTORC1-mediated overactivation of the kinase S6K1 [277], which via inhibitory phosphorylation of insulin receptor substrate 1 (IRS-1) attenuates insulin signaling [286,287,288,289].

### 3.4. Diabetes Mellitus Type 2

Insulin resistance has been observed in children after high consumption of milk compared to meat intake [290]. The first meta-analysis, which investigated the effects of milk versus fermented milk and their relation to diabetes mellitus type 2 (DMT2), is the European Prospective Investigation into Cancer and Nutrition (EPIC) [291]. EPIC shows an increased risk of DMT2 by milk consumption in five out of eight European countries including Germany [291]. The Framingham Heart Study Offspring Cohort [292] and the Physicians´ Health Study [293] confirm an association between milk consumption and prediabetes as well as DMT2. The Dutch Lifeline Cohort Study exhibits a positive association between whole milk intake (150 g/day) and prediabetes as well as a relation between milk consumption (150 g/day), especially skim milk (150 g/day) and DMT2 [294]. Increased β-cell mTORC1 activity plays a critical role in the pathogenesis of DMT2 [295,296,297,298,299,300,301,302], which is normalized by the AMPK activator and mTORC1 inhibitor metformin [303,304,305]. Recent evidence underlines that the ratio mTORC1/AMPK is increased in DMT2 [301]. Pancreatic β-cells differentiate during fetal life, but only postnatally acquire the capacity for glucose-stimulated insulin secretion (GSIS) [301]. An increased responsiveness to dietary glucose is required at the end of the breastfeeding period, which delivers a nearly constant supply of lactose. The dramatic change during weaning is associated with a switch of increased mTORC1 activity to enhanced AMPK activity. While mTORC1 signaling stimulates β-cell proliferation, AMPK signaling promotes β-cell mitochondrial biogenesis, a shift to oxidative metabolism, and functional maturation of β-cells [301]. In fact, increased plasma levels of miR-148a have been associated with T2DM progression, increased HbA1c, HOMA-IR, and hyperinsulinemia [306]. It has been hypothesized that MEX miR-148a, which inhibits AMPK, maintains the hyperactivated state of β-cell mTORC1 activity during the period of breastfeeding (milk intake), a meaningful mechanism during the breastfeeding period, but a detrimental effect for β-cell homeostasis in the long-run [302].

### 3.5. Prostate Cancer

The Physicians’ Health Study identified the consumption of whole milk as a risk factor of prostate cancer (PCa) [293]. The meta-analysis of Lu et al. [307] confirmed a dose-dependent correlation between milk consumption and PCa mortality. The Reykjavik Study showed that daily milk consumption during the first 20 years of life compared to non-daily milk intake increased the risk of advanced PCa by the factor of 3.2 [308]. In analogy to acne [276,277,278,279,280,281], increased IGF-1/PI3K/AKT/mTORC1 signaling plays a key role in prostate morphogenesis and cancerogenesis [309,310,311,312,313,314]. S-adenosylmethionine decarboxylase 1 (AMD1) is upregulated in human PCa dependent on activated mTORC1 [315]. In PCa tissue, mTORC1 integrates and translates growth signals into an oncogenic metabolic program [311,312,313,314,315,316]. Notably, the activity of the lipogenic transcription factor SREBF1, which is important for PCa cell growth, is regulated by androgen receptor (AR)/mTOR nuclear axis in PCa [317]. Increased recurrence rates and more aggressive courses correlate to the consumption of whole milk, but not fermented milk products [318,319].

Data from molecular pathology support the involvement of miR-148a, miR-125b, and miR-21 in PCa initiation and progression. MiR-148a is upregulated in serum and PCa tissue of men with PCa associated with an increase in Gleason score [320]. MiR-148a is an androgen-responsive miR that promotes LNCaP prostate cell growth by repressing its target cullin-associated and neddylation-dissociated 1 (CAND1). CAND1 is a negative regulator of SKP1-Cullin1-F-box (SCF) ubiquitin ligases by binding to the 3′-untranslated region of CAND1 mRNA [321], thereby increasing cellular levels of cyclin E [322]. Interestingly, cyclin-dependent kinase inhibitor 1B (CDKN1B) loss is an important initiator of PCa [323]. MiR-148a silences CDKN1B, which is consistent with the increase in proliferation through increased S-phase transition observed after miR-148a transfection into LNCaP cells [324]. In addition, miR-148a inhibits DNMT1 [132,149,169], which enhances AR expression via the repressive E2F1/DNMT1 axis [325]. Furthermore, miR-148a promotes DNMT1-dependent epithelial-mesenchymal transition (EMT) and the progression of PCa stem cells [326]. MiR-148a-mediated suppression of AMPK, which increases mTORC1 activation [104,105], appears to be the rationale for the treatment and prevention of PCa with AMPK agonists/mTORC1 antagonists [303,327,328].

MiR-125b plays an emerging role in several cancers [329] and is an androgen-induced miR that is overexpressed in PCa [330,331]. MiR-125b promotes growth of PCa xenograft tumor through targeting proapoptotic genes including p53, PUMA, and BAK1 [332]. MiR-125b targets p14 affecting p14(ARF)/MDM2 resulting in enhanced p53 catabolism with proliferation of PCa cells [333]. MiR-125b is involved in regulating NF-κB, p53, PI3K/AKT/mTORC1, ERBB2, WNT, and other signaling pathways, thereby controlling cell proliferation, differentiation, metabolism, apoptosis, drug resistance, and tumor immunity. MiR-21 is another oncogenic miR overexpressed in PCa that inhibits various tumor suppressor genes such as PTEN enhancing mTORC1 activation [334,335,336,337,338,339,340,341,342,343,344].

Thus, MEX-derived circulatory miRs that may reach the prostate and target critical tumor suppressor genes enhance the activity of mTORC1 [207,312].

### 3.6. Breast Cancer

The Norwegian Cancer Registry showed that daily intake of >750 mL whole milk compared to <150 mL daily milk intake enhanced the risk of BC by the factor of 2.91 [345]. McCann et al. [346] observed positive associations between milk intake and risk of estrogen receptor (ER)-negative BC (OR: 1.58; 95% CI: 1.05, 2.37). Frazer et al. [347] reported an increase in BC risk (HR = 1.50; 95% CI: 1.22–1.84) related to milk consumption independent of milk fat content in a Californian cohort. A stronger association was found in ER+ and progesterone receptor-positive (PR+) tumors. Already the daily intake of 158 mL milk enhanced BC risk, whereas the consumption of cheese and yogurt had no negative effects. Kaluza et al. [348] recently confirmed, that high and continuous consumption of two daily servings of non-fermented milk compared to no milk consumption, increased the incidence of ER+/PR+ BCs (HR = 1.30; 95% CI: 1.02–1.65). Especially women with normal weight (BMI < 25 kg/m^2^) exhibited an increased BC risk (HR = 1.55; 95% CI: 1.08–2.21). In contrast, the consumption of fermented milk products showed an inverse incidence in ER/PR-cancers [193]. A Chinese study identified high milk consumption as a BC risk factor in women of rural regions [349]. Further controlled case studies show that milk consumption increases BC risk [350,351], whereas lactose intolerance reduces BC risk [352].

Early menarche, elevated IGF-1 serum levels, increased breast density, and accelerated breast growth during adolescence are well-known risk factors of BC [353,354,355,356,357,358,359]. The intake of sugar-based milk drinks (>125 g/day) increased fibroglandular volume in girls [360]. IGF-1 and estrogens, which are increased in milk of persistently pregnant dairy cows [361], have a synergistic effect in mammary cancerogenesis [362,363].

Essential PI3K/mTORC1 signaling pathway activation has been observed in most BCs [364,365,366,367,368,369]. Mutations in the PI3K/AKT/mTOR pathway are frequently found in BC and associated with cellular transformation, tumorigenesis, cancer progression, and drug resistance [365,366,367,368,369]. In particular, hyperactivation of the PI3K/AKT/mTORC1 is implicated in the tumorigenesis of ER+ BC and in resistance to endocrine therapy [364,368,369]. Metformin in a diabetes-independent manner significantly reduces BC risk, compared to patients who are not using metformin [370]. Kelch-like 22 (KLHL22) is a broad complex, tramtrack, and bric-a-brac (BTB) adaptor protein, which forms a functional cullin-RING E3 ubiquitin ligase complex with the scaffold protein CUL3 and the ring finger protein RBX1, is upregulated in BCs. Independent of PI3K/AKT signaling, KLHL22 activates amino acid-dependent mTORC1 signaling to promote tumorigenesis [371].

Importantly, mTORC1 emerged as a critical node in estrogenic signaling in BC cells. Estrogen rapidly and potently activates mTORC1 signaling. In addition, mTORC1 is a crucial activator of ERα transcriptional activity [372]. ERα binds to Raptor and causes it to translocate to the nucleus upon estrogen stimulation. In addition, nuclear mTOR kinase phosphorylates ERα on S104/106 and thereby activates transcription of ER target genes [373]. Upon mitogen and estrogen stimulation, S6K1 and mTORC1, respectively, are able to phosphorylate ERα, significantly affecting chromatin binding and transcriptional activity in a ligand independent fashion [373,374,375,376], while establishing a feed-forward mechanism that may drive cancer progression through upregulation of eIF3 by ERα [377,378].

Of note, the biological effects of estrogens are mainly mediated by the activation of ERα, whose activity is deeply influenced by the insulin/IGF-I signaling pathway [379,380]. In fact, IGF-1-mediated activation of S6K1 promotes ERα (S167) phosphorylation affecting the transcription of ERα-regulated genes [381]. Thus, increased insulin/IGF-1 signaling by consumption of commercial milk increases ERα-induced gene expression. There is recent evidence that ERα signaling is also upregulated by miRs [382], especially miR-148a [383], which functions as an oncogene of tumor suppressor depending on the cancer type [384]. miRNA-148a targets DNMT1, which suppresses the expression of ER-α via DNA methylation [383]. Indeed, miRNA-148a regulates ER-α expression through DNMT1-mediated DNA methylation in BC cells, whereas miRNA-148a inhibition decreases ERα expression [383]. ERα enhances the expression of L-type amino acid transporter 1 (LAT1, SLC7A2 [385,386,387], which increases cellular leucine influx activating mTORC1 [73,74,75,76,77,78,79,80,81,82,83,84]. In fact, increased expression of LAT1 has been reported in BC [388,389], preferentially in chemoresistant BC [390]. Of note, proliferation-related genes are highly expressed in a subgroup of patients with high SLC7A5/SLC3A2, and knockdown of SLC7A5/SLC3A2 decreased proliferation of ER+ BC cells [391]. Saito et al. [392] found that scribble cell polarity complex component 2 (LLGL) regulates proliferation of ER+ BC cells in culture and in vivo under leucine stress conditions by promoting uptake of leucine. LLGL2 interacts with SLC7A5 by forming a trimeric complex with SLC7A5 and a regulator of membrane fusion, YKT6, to promote leucine uptake and cell proliferation [392].

MiR-21, a component of bovine MEX [209,393], is overexpressed in BC compared with normal breast tissue and has been associated with advanced stage, lymph node positivity, and reduced survival time [394,395,396]. Of note, increased levels of circulating miR-21 in plasma and plasma exosomes has been reported in BC patients [397,398,399].

In accordance with miR-21, both plasma and tissue expression of miR-155 increased in BC patients compared to controls [400]. Importantly, miR-155-enriched exosomes accelerate EMT of BC cells [401]. Interestingly, functional BRCA1 inhibits the expression of oncogenic miR-155 [402,403]. Individuals with BRCA1 loss-of-function mutations are thus exposed to higher oncogenic signaling of miR-155, which may be further aggravated by the uptake of MEX-derived miR-155.

Remarkably, increased miR-155 expression in the ERα+ BC cells results in a repression of RICTOR enhancing activation of mTORC1 signaling [404]. Furthermore, FBXW7, which promotes mTOR degradation [203,405], is less expressed in BC tissues and cell lines, and is an independent positive factor for the overall survival rate of patients with BC [406,407]. MiR-155 and miR-223, abundant components of cow milk [138,139,163,198,199,203], both target FBXW7 and thus increase mTORC1 signaling [202,408,409].

Taken together milk-derived miRs may enhance mTORC1 signaling in BC cells at various checkpoints of the mTORC1 signaling cascade.

### 3.7. Hepatocellular Carcinoma

EPIC demonstrated an association of increased risk of hepatocellular carcinoma (HCC) with the consumption of milk (HR = 1.51; 95% CI: 1.02–2.24), but not yogurt (HR = 0.94, 95% CI: 0.65–1.35) [410]. The Health Professionals Follow-Up Study and Nurses’ Health Study confirmed an increased risk of HCC with milk consumption (HR = 1.23; 95% CI: 0.83–1.83), especially skim milk (HR = 1.36; 95% CI: 0.91–2.03), but not yogurt (HR = 0.72; 95% CI: 0.49–1.05) [411]. The Guangzhou Biobank Cohort Study showed that moderate milk intake (250–750 mL/week) compared with higher milk consumption (>750 mL/week) was associated with an increase of all cancer mortality and enhanced HCC mortality from 3.8 to 7.0 per 10,000 person years [412].

In HCC tissue, mTORC1 is highly activated [413,414,415,416]. Loss of BCAA catabolism during carcinogenesis enhances mTORC1 activity and promotes tumor development and progression [414,415]. MEX-derived miR-29b via targeting BCKD [225], may promote HCC [417]. As shown in mice after oral administration of bovine MEX, MEX and miR-21 accumulate in the liver and other tissues [134,209]. Plasma exosomal miR-21 and miR-155 are oncogenic miRs promoting mTORC1-driven hepatocancerogenesis [417,418,419,420,421,422,423]. Thus, upregulated mTORC1 via milk-derived BCAAs and oncogenic MEX-derived miRs may explain the increased risk of HCC by milk consumption [417].

### 3.8. Diffuse Large B-Cell Lymphoma

According to a large meta-analysis [424], a positive association has been reported for milk consumption and non-Hodgkin’s lymphoma (NHL) (RR = 1.41; 95% CI: 1.08–1.84), whereas a reduced risk was observed for yogurt consumption (RR = 0.78; 95% CI: 0.54–1.12). Each 200 g of daily milk intake increased the risk of NHL by 6% [424]. After NHL subtype differentiation, a significant association was found between milk intake and diffuse large B-cell lymphoma (DLBCL) (RR = 1.49; 95% CI: 1.08–2.06). DLBCL is the most common type of lymphoma, representing approximately one-third of all cases worldwide [425].

In DLBCL, mTORC1 signaling is upregulated [426,427] and is therapeutically attenuated by the mTORC1 inhibitor everolimus [427]. MiR-21 as well as miR-155 promote the proliferation of malignant B-lymphocytes [428,429,430,431,432,433,434,435]. Of note, miR-21 plays an oncogenic role by targeting FOXO1 and activating the PI3K/AKT pathway in DLBCL [429]. Overexpression of plasma miR-155 was significantly upregulated in patients with DLBCL compared to healthy individuals and was related to a shorter overall survival time [436]. B-cell lymphoma cells showed a higher expression of miR-155 and a low expression of FOXO3 than B-lymphocytes [437]. FOXO3-mediated expression of sestrin 3 activates AMPK [438], which via TSC2 phosphorylation inhibits mTORC1 [439]. Reduced FOXO1 and FOXO3 expression via overexpression of miR-21 and miR-155, respectively, thus increase mTORC1 signaling in DLBCL lymphocytes.

### 3.9. Parkinson’s Disease

The Greek EPIC cohort showed a significant correlation between milk consumption and Parkinson’s disease (PD) (HR = 1.34; 95% CI: 1.14–1.58), whereas cheese and yogurt consumption showed no association [440]. A large meta-analysis of prospective cohort studies identified an increased risk for PD by milk consumption (RR = 1.45; 95% CI: 1.23–1.73), cheese (RR = 1.26; 95% CI: 0.99–1.60), but not yogurt (RR = 0.95; 95% CI: 0.76–1.20) [441]. The Nurses’ Health Study and the Health Professionals Follow-up Study confirmed an increased risk of PD with consumption of low-fat milk (HR = 1.39; 95% CI: 1.12–1.73) and milk of all fat levels (HR = 1.56; 95% CI: 1.30–1.88) [442]. Olsson et al. [443] studied the influence of milk versus fermented milk in Swedish PD patients. Compared to no or low milk intake (<40 mL/day), milk consumption of 40–159 mL/day showed a HR = 1.29 (95% CI: 1.07–1.56), 160–200 mL/day a HR = 1.19 (95% CI: 0.99–1.42), 201–400 mL/day a HR = 1.29 (95% CI: 1.08–1.53), and over 400 mL/day a HR = 1.14 (95% CI: 0.93–1.40). Fermented milk was not associated with PD risk [443].

The hypothesis that contamination of milk with neurotoxic compounds is causal for milk’s PD-inducing effects [444] has recently been challenged [445]. There is accumulating evidence that milk’s intrinsic mTORC1-activating signaling capacity promotes the pathogenesis of PD [445]. PD is an α-synucleinopathy associated with mitochondrial dysfunction, oxidative stress, deficient lysosomal clearance of α-synuclein (α-syn), and aggregation of misfolded α-syn [446,447,448]. Increasing evidence substantiates that imbalances of mTORC1 and autophagy are critically involved in the pathogenesis of PD [449,450,451,452]. Enteroendocrine cells, which are able to synthesize α-syn and exhibit vagal nerve connectivity to the brain, are in the recent focus in PD pathogenesis [453,454,455,456,457,458,459]. In contrast to milk consumption, increased intake of caffeine and green tea polyphenols and smoking have been associated with a decreased risk of PD [460]. Remarkably, caffeine, epigallocatechin-3-gallate, and nicotine are inhibitors of mTORC1 activating autophagy [461,462,463,464,465,466]. Milk via activation of mTORC1 may inhibits ULK-1, the key mediator of mTORC1 signaling to autophagy, that regulates early stages of autophagosome formation in response to starvation or mTORC1 inhibition [467].

Notably, hypomethylation of the SNCA promoter increases α-syn expression, which is controlled by DNMT1 [468,469,470,471,472]. Intriguingly, the neurotoxic compound 1-methyl-4-phenyl-1,2,3,6-tetrahydropyridine (MPTP), which is used in murine models for the induction of PD, increases the expression of miR-148a associated with downregulation of DNMT1 in substantia nigra of MPTP-treated mice [473]. It is conceivable, that MEX miR-148a targets DNMT1 expression of enteroendocrine cells increasing the expression of α-syn [445]. Moreover, AMPK-induced autophagy may be further attenuated by MEX miR-148a. It has been demonstrated that the upregulation of miR-148a inhibits the expression of AMPK [187], resulting in increased mTORC1 activity [104] and attenuated ULK1-mediated autophagy [474,475,476].

In addition, increased expression of miR-21 has been reported in substantia nigra of PD patients associated with decreased expression of lysosome-associated membrane protein type 2A (LAMP2A), which is a direct target of miR-21 [477,478]. LAMP2A plays a key role in chaperone-mediated autophagy (CMA), which is disturbed in PD [468,478].

Thus, milk signaling via MEX-derived miRs may overactivate mTORC1 and decrease autophagy resulting in overexpression of α-syn and impaired degradation of aggregated neurotoxic α-syn promoting the pathogenesis of PD.

### 3.10. Alzheimer’s Disease

Epidemiological studies on milk consumption and Alzheimer’s disease (AD) and cognitive decline are contradictory. According to a systematic review and meta-analysis, Lee et al. [479] concluded that the existing evidence is too poor to draw a firm conclusion regarding the effect of milk or dairy intake on the risk of cognitive decline or disorders in adults. However, Kesse-Guyot et al. [480] reported that milk intake but not total dairy was negatively associated with verbal memory performance. Furthermore, Petruski-Ivleva et al. [481] have studied 13,751 participants of the Atherosclerosis Risk in Communities (ARIC) cohort and performed three neurocognitive evaluations from 1990 through 2013. They observed that milk intake greater than 1 glass/day was associated with greater decline in cognitive functions over a 20-year observation period. Despite the scarcity of evidence on this topic, the latest systematic review on milk and dairy intake points to a cognitive decline associated with milk consumption [482].

AD is now the most common form of neurodegenerative dementia in the United States and other Western countries [483]. Subsequent progressive changes in cognition and behavior accompany the later stages of AD. Changes in amyloid precursor protein (APP) cleavage and production of the APP fragment β-amyloid (Aβ), along with hyperphosphorylated tau protein aggregation coalesce to cause reduction in synaptic strength, synaptic loss, and neurodegeneration [484,485]. AD is characterized by the presence of two aberrant structures: namely senile plaques, composed of amyloid-β peptide (Aβ), and neurofibrillary tangles, composed of tau protein [486,487]. AD thus belongs to the group of tauopathies associated with accumulation of abnormal tau protein in the brain [486,487,488,489]. Phosphorylation of different tau sites during progression of AD been reported [490]. Substantial evidence indicates that mTORC1 is involved in the formation, secretion, and degradation of toxic phospho-tau [491,492,493,494]. The hyperphosphorylation of tau protein and the overexpression of mTORC1 are considered the driving force behind Aβ plaques and neurofibrillary tangles, hallmarks of AD [495]. Norambuena et al. [496] reported a crosstalk between mitochondria and lysosomes and identified a role for lysosomal mTORC1 in the nutrient-induced activation of mitochondria. This lysosomal signaling pathway is strongly inhibited by oligomeric Aβ through the tau-dependent activation of plasma membrane-localized mTORC1. Together, these results identify a further role for tau in mediating Aβ toxicity [497]. A number of mTORC1-dependent and independent autophagy modulators have been identified to have positive effects in AD treatment [498,499]. Recent evidence indicates that mTORC1 inhibition and autophagy activity are directly linked to tau clearance [500]. In contrast to neuronal mTORC1 signaling, microglial deficiency of TREM2, a surface receptor required for microglial responses to neurodegeneration, including proliferation, survival, clustering, and phagocytosis, has been associated with impaired mTORC1 activity and anomalous autophagy [501].

The microtubule-associated protein tau (MAPT) has been identified in several intraneuronal compartments, including in association with synapses [502,503]. Tau is a microtubule-associated protein that has a role in stabilizing neuronal microtubules and promotes axonal outgrowth. Structurally, tau is a natively unfolded protein, is highly soluble and shows little tendency for aggregation [504]. In analogy with the epigenetic regulation of the *SNCA* promoter in PD, increased tau expression is induced by decreased *MAPT* promoter methylation [505,506]. It has been demonstrated that DNMT1 is an epigenetic regulator of MAPT expression [507]. In contrast, hypermethylation of the *MAPT* gene is neuroprotective by reducing MAPT expression [508].

During the breastfeeding period with physiological transfer of MEX and MEX-derived miR-148a and miR-21 to neuronal cells, miR-148a/miR21-mediated DNMT1 suppression may enhance overall SNCA and MAPT expression for postnatal maturation of synapses promoting synaptic connectivity, in accordance with observed improvements of cognitive functions in mice receiving a MEX-sufficient diet compared to a MEX-deficient diet [509]. Beneficial effects of breastfeeding and cow milk-mediated epigenetic regulation in early life may thus turn into adverse effects when milk signaling is not discontinued, as originally programmed by mammalian physiology.

Dysfunction of cell bioenergetics is a common feature of neurodegenerative diseases, the most common of which is AD [510,511] promoting synaptic transmission failure [512]. Oxidative stress is a key driver promoting dysfunction of mitochondria, which are vulnerable to oxidative stress [513,514,515]. D-Galactose, the hydrolysis product of the milk sugar lactose, is a well-known mitochondrial stressor experimentally used for the induction of brain aging and neurodegeneration [124,516,517,518,519,520,521,522,523,524,525,526]. In humans, hepatic galactose clearance declines with age [519,520,521]. Notably, galactose induces oxidative stress activating mTORC1 [124] and increases the expression of miR-21 [522].

MiR-148a targets *PPARGC1A* (peroxisome proliferator-activated receptor-γ coactivator-1α, PGC-1α) [523] (targetscan.org, accessed on 16 February 2021), which is a key transcriptional regulator in tissues that undergo extensive oxidative metabolism and operates as a central organizer of metabolic function, oxidative states, and mitochondrial biogenesis and function [524]. PGC-1α cooperates with estrogen-related receptor-α (ERRα) in the regulation of mitochondrial biogenesis [525] and plays a central role in the regulation of autophagy [526].

Taken together, persistent milk signaling apparently stimulates overexpression of tau proteins as well as mTORC1-mediated tau phosphorylation promoting the formation of neurofibrillary tangles, enhances galactose-mediated oxidative stress as well as miR-148a-mediated mitochondrial dysfunction and impaired autophagy, all pathological hallmarks of AD.

## 4. Fermentation, All-Cause Mortality, and Aging

Four epidemiological studies from Sweden, a country with high per capita milk consumption of pasteurized fresh milk, underline an increased dose-dependent risk of all-cause mortality with the consumption of milk [527,528,529,530,531], but not fermented milk/milk products [528,531,532].

Since the Neolithic revolution, the great majority of milk was consumed as fermented milk and fermented milk products [533,534,535]. However, an unnoticed dramatic change occurred with the introduction of pasteurization and refrigeration of milk, which preserved milk’s bioactive exosomal miRs [132,133,134,135], allowing them to enter the human food chain in large-scale [170,171]. Pasteurization thus preserves milk’s bioactive mTORC1 activators including galactose, essential amino acids, and exosomal miRs [132,135,145,160,198,527], whereas fermentation degrades galactose [536,537,538,539], essential branched-chain amino acids [540,541], MEX and their miRs, respectively [393]. Whereas addition of milk to a meal increases postprandial insulin levels [542], addition of yogurt reduces postprandial insulinemia [53], thus reduces insulin-mediated mTORC1 signaling. Further information on the impact of fermentation versus pasteurization of milk has been presented elsewhere [9].

**Figure 3:**
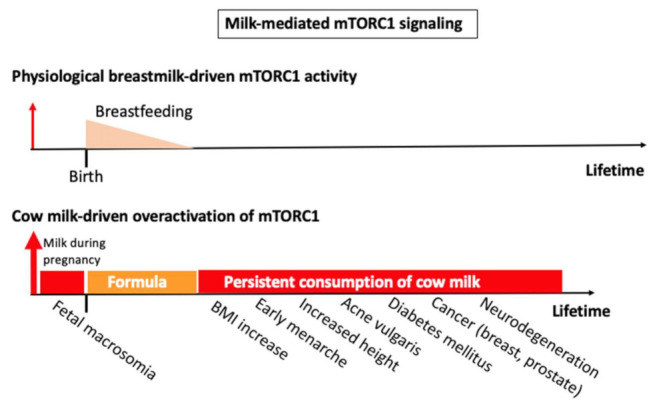
Milk-mediated mTORC1 signaling. Upper panel: physiological milk signaling exclusively only during the postnatal breastfeeding period with milk derived from the biological mother (human lactation genome). Lower panel: cow milk-driven overactivation of mTORC1 begins with maternal cow milk consumption during pregnancy, continues with high protein cow milk-based artificial formula, and continues with milk consumption during all age periods of human life. Persistent milk signaling with overactivated mTORC1 modifies growth trajectories during childhood and adolescence and promotes diseases of civilization.

Notably, recent evidence underlines that mTORC1 activates the expression of RNA polymerase III (Pol III), which limits longevity [543]. Increased mTORC1 signaling shortens lifespan and accelerates aging-related processes such as cellular senescence and stem cell exhaustion [544,545,546,547,548,549,550,551,552,553,554,555]. Thus, persistent overactivation of mTORC1 by continued cow milk consumption accelerates aging and overall mortality of mTORC1-driven diseases of civilization (Figure 3).

## 5. Conclusions

Milk, the secretory product of mammary glands, executes the species-specific genetic program of the lactation genome. Milk should not be regarded as a “simple food”, but it instead represents the signaling interface between the maternal lactation genome and the infant’s cellular mTORC1 system orchestrating growth, anabolisms, metabolic, immunological, and neurological programming [6]. Milk is the exclusive nutrient and nutrigenetic offer for newborn mammals sufficient and well adapted to promote adequate mTORC1-dependent postnatal growth [7]. Obviously, milk presents the masterpiece of mammalian evolution to assist for extrauterine growth, optimized during millions of years of lactation evolution [128]. Thereby, milk relays sophisticated maternal signals for mTORC1 activation to the milk receiver. Milk’s amino acids directly activate mTORC1 via the Rag-Ragulator pathway. Whey- and casein-derived amino acids promote insulin and IGF-1 secretion, respectively, increasing PI3K-AKT-mediated activation of mTORC1. Milk amino acid-mediated activation of mTORC1 is supported by a complex network of exosomal miRs that epigenetically enhance mTORC1 signaling [153].

With the introduction of pasteurization (72 °C, 15 s) combined with refrigeration, the human milk consumer got exposed to bioactive MEX miRs augmenting milk’s mTORC1 activity compared to boiled, ultra-heat-treated (UHT), or fermented milk. The high conservation of milk miRs among various mammals underlines the importance of MEX-derived miRs in the epigenetic regulation for postnatal mammalian growth [153,165]. Notably, among all mammals, only humans experience life-long exposure to cow milk signaling overactivating mTORC1 (Figure 3).

Milk consumption during pregnancy already promotes fetal overgrowth via abundant supply of essential amino acids and placental transfer of bovine MEX and their miRs [209,239,240,241,242,243]. Milk accelerates BMI [258], early onset of menarche [251], skeletal growth and height [42,256,257], sebaceous gland hyperplasia, and sebaceous lipogenesis promoting mTORC1-driven acne vulgaris [274,277,278]. Milk consumption during adult life is associated with higher risks of common mTORC1-driven cancers, including PCa [307,308,309,310,311,312,313,314,315,316], BC [345,346,347,348,349], HCC [410,411,417], DLBCL [424], and promotes the neurodegenerative diseases PD [440,441,442,443,444,445] and AD [480,481,482], which are all related to overstimulated mTORC1 signaling (Figure 3). Thus, milk’s physiological function to maintain high mTORC1 signaling at the beginning of mammalian life turns into adverse health effects when this postnatal endocrine and epigenetic system is not discontinued as designated by the physiological processing of the lactation genome. A deeper understanding of milk’s interaction with the central hub of metabolic regulation, mTORC1, will open new avenues for prevention of common diseases of civilization.

## Figures and Tables

**Table 1:** Amino acid composition of milk proteins compared to aninmal- and plant-based protein sources (g amino acids/100 g protein).

Amino acid	Milk	Casein	Whey	Cod	Chicken	Egg	Beef	Pork	Lentil	Bean	Soy
Leucine	10.4	10.4	11.1	8.28	7.41	8.4	8.09	7.61	9.02	8.35	7.34
Isoleucine	6.4	5.7	6.8	4.65	5.43	6.22	4.98	4.95	5.08	4.55	4.66
Valine	6.8	6.8	6.8	5.34	5.06	7.48	5.43	5.62	5.94	5.12	4.61
Tryptophan	1.4	1.4	2.1	1.18	1.3	1.51	1.12	1.19	1.07	0.99	1.18
Methionine	2.8	2.9	2.2	2.94	2.67	3.03	2.47	2.79	0.94	1.24	1.52
Arginine	3.7	4	3	5.93	6.48	5.97	5.99	5.97	9.57	6.36	6.181
Glutamine ^*^	8.1	n.d.	n.d.	n.d.	n.d.	4.43	4.75	n.d.	n.d.	n.d.	9.14

Amino acid data according to [66] and glutamine data * [68]; n.d.= not determined.

## Data Availability

All-data are derived from reviewed publishes references listed in the PubMed data base.

## Abbreviations

Aβ	β-amyloid
AD	Alzheimer’s disease
AMD1	S-adenosylmethionine decarboxylase 1
AMPK	adenosine monophosphate-activated protein kinase
APP	amyloid precursor protein
AR	androgen receptor
ARF1	ADP-ribosylation factor 1
ARIC	Atherosclerosis Risk in Communities
BAK1	BCL2 antagonist killer 1
BC	breast cancer
BCAA	branched-chain amino acid
BCKD	branched-chain alpha-ketoacid dehydrogenase
BMI	body mass index
BRCA1	breast cancer 1 protein
BTB	broad complex, tramtrack, and bric-a-brac
CAND1	cullin-associated and neddylation-dissociated 1
CASTOR1	cellular arginine sensor for mTORC1
CDKN1B	cyclin-dependent kinase inhibitor 1B
CMA	chaperone-mediated autophagy
CUL3	cullin 3
DBT	dihydrolipoamide branched-chain transacylase
DLBCL	diffuse large B-cell lymphoma
DMT2	diabetes mellitus type 2
DNMT	DNA methyltransferase
eIF4e	eukaryotic translation initiation factor 4E
GAP	GTPase-activating protein
E2F1	E2F transcription factor 1
EMT	epithelial-mesenchymal transition
EPIC	European Prospective Investigation into Cancer and Nutrition
ER	estrogen receptor
ERBB2	ERB-B2 receptor tyrosine kinase 2
ERRα	estrogen-related receptor alpha
ER stress	endoplasmic reticulum stress
EV	extracellular vesicle
FBP	fructose-1,6-bisphosphate
FBXW7	F-box and WD40 domain protein 7
FOXO	forkhead box transcription factor
FTO	fat mass- and obesity-associated gene
GH	growth hormone
GHR	growth hormone receptor
GHRH	growth hormone releasing hormone
GSIS	glucose-stimulated insulin secretion
HCC	hepatocellular carcinoma
HR	hazard ratio
HT	hydroxytryptamine
IGF-1	insulin-like growth factor 1
IGF1R	IGF-1 receptor
IGFBP3	IGF binding protein 3
IRS1	insulin receptor substrate 1
KLHL22	Kelch-like 22
LAMP2A	lysosome-associated membrane protein type 2A
LAT1	L-type amino acid transporter 1; SLC7A5
LCT	lactase gene
LKB1	serine/threonine protein kinase 11
LRS	leucyl-tRNA synthetase
MAPT	microtubule-associated protein tau
MDM2	mouse double minute 2 homolog
MEX	milk exosome
MFG	milk fat globule
MFG-E8	MFG EGF-factor 8
miR	micro-ribonucleic acid
MPTP	1-methyl-4-phenyl-1,2,3,6-tetrahydropyridine
mTORC1	mechanistic target of rapamycin complex 1
NF-κB	nuclear factor kappa B
NHANES	United States National Health and Nutrition Examination Survey
NHL	non-Hodgkin lymphoma
NRF2	nuclear factor erythroid 2-related factor 2
PCa	prostate cancer
PCR	polymerase chain reaction
PD	Parkinson’s disease
PDCD4	programmed cell death 4
PI3K	phosphoinositide-3 kinase
PPARGC1A	peroxisome proliferator-activated receptor-γ coactivator-1α, PGC-1α
PR	progesterone receptor
PRKAA1	catalytic subunit α1 of AMPK
PRKAG2	regulatory subunit γ2 of AMPK
PTEN	phosphatase and tensin homolog
PUMA	p53-upregulated modulator of apoptosis; BBC3
RAG	Ras-related GTP-binding protein
Raptor	regulatory associated protein of mTOR
RHEB	Ras-homolog enriched in brain
SAMTOR	S-adenosylmethionine sensor upstream of mTOR
SESN2	sestrin 2
S6K1	ribosomal protein S6 kinase, 70-KD, 1;
SLC3A2	solute carrier family 3, member 2
SLC7A5	solute carrier family member 5; LAT1
SNCA	synuclein, alpha
SREBF1	sterol regulatory element binding transcription factor 1
TAG	triacylglycerol
TP53	tumor protein p53
TSC2	tuberin
UHT	ultra-heat-treated
ULK1	unc-51 like autophagy activating kinase 1
WHO	World Health Organization
